# Supplementary material for: MitoZ: a toolkit for animal mitochondrial genome assembly, annotation and visualization
Source: Nucleic Acids Res. 2019 Mar 13;47(11):e63. doi: 10.1093/nar/gkz173 (PMC6582343; doi:10.1093/nar/gkz173)
Supplement: gkz173_Supplemental_Files [file gkz173_supplemental_files.zip › Supplementary Tables.docx]

**Table S1. Species list used for MitoZ performance testing**

**Table S2. A+T content of sanger mitogenome and sanger mitochondrial genes, mitochondrial reads ratio and heterozygosity of SRA data**

**Table S3. Procedures and species list for selecting test species for arthropods**

**Table S4. Procedures and species list for selecting test species for mammals**

**Table S5. Species list for building PCGs HMM models**

**Table S6. Species list for building mitochondrial PCGs annotation database**

**Table S7. MitoZ genes vs. sanger genes of 29 arthropod samples**

**Table S8. MitoZ genes vs. sanger genes of 21 mammal samples**

**Table S9. Information of the five false positive genes and samples with low similarity (<97%) genes in MitoZ assembly**

**Table S10.** **NOVOPlasty genes vs. sanger genes of 29 arthropod samples**

**Table S11. NOVOPlasty genes vs. sanger genes of 21 mammal samples**
